# Transcription and Post-translational Regulation of Autophagy in Insects

**DOI:** 10.3389/fphys.2022.825202

**Published:** 2022-02-25

**Authors:** Rongsong Li, Yang Xiao, Kang Li, Ling Tian

**Affiliations:** ^1^Guangdong Provincial Key Laboratory of Agro-animal Genomics and Molecular Breeding, College of Animal Science, South China Agricultural University, Guangzhou, China; ^2^Guangdong Laboratory for Lingnan Modern Agriculture, Guangzhou, China; ^3^Guangdong Provincial Sericulture and Mulberry Engineering Research Center, College of Animal Science, South China Agricultural University, Guangzhou, China; ^4^Department of Sericulture and Southern Medicine Resources Utilization, The Sericultural and Agri-Food Research Institute of the Guangdong Academy of Agricultural Sciences, Guangzhou, China; ^5^Guangdong Provincial Key Laboratory of Insect Developmental Biology and Applied Technology, Institute of Insect Science and Technology, School of Life Sciences, South China Normal University, Guangzhou, China

**Keywords:** autophagy, transcription, post-translational modification, insect hormone, nutrient signal

## Abstract

Autophagy attracts great attention, and numerous progresses have been obtained in the last two decades. Autophagy is implicated in mammalian neurodegenerative diseases, tumorigenesis, as well as development in insects. The regulatory mechanism of autophagy is well documented in yeast and mammals, whereas it is not fully illustrated in insects. *Drosophila melanogaster* and *Bombyx mori* are the two well-studied insects for autophagy, and several insect-mammalian evolutionarily conserved or insect-specific mechanisms in regulating autophagy are reported. In this review, we summarize the most recent studies of autophagy regulated at both transcriptional and post-translational levels by insect hormone in cooperation with other signals, such as nutrient, which will provide a reference and deep thinking for studies on autophagy in insects.

## Introduction

Macroautophagy, hereafter referred to as autophagy, is a self-eating process to recycle intracellular components and extensively exists in organisms under physiological and pathophysiological conditions (Klionsky et al., 2021). Autophagy is associated with development, pathogenicity, tumorigenesis, and neurodegenerative diseases (Wang et al., 2020a; Klionsky et al., 2021). During tumorigenesis, autophagy is evoked to remove oncogenic protein substrates and toxic-unfolded proteins, while autophagy flux usually facilitates the spreading of tumor cells after the formation (Wang et al., 2020a). In the process of autophagy occurrence, the cytoplasmic constituents (usually contains misfolded proteins and damaged organelles) are isolated and packaged by double-membraned phagophores, which grow to form the sphere-like autophagosomes. After maturation, autophagosome fuses with lysosome to form autolysosome for bulk degradation of the cargoes by hydrolytic enzymes inside lysosomes (Klionsky et al., 2021; Wang et al., 2021a). Formation of autophagosomes recruits a series of autophagy-related (Atg) proteins, participating in the steps of induction, cargo identification and packaging, as well as maturation and degradation of autophagosomes (Klionsky et al., 2021). Some *Atg* genes have been identified in *Drosophila melanogaster* and *Bombyx mori*, whereas not all of the homologs revealed in yeast and mammals are found in insects (Tian et al., 2013; Guo et al., 2019).

Autophagy is involved in certain physiological processes in insects including development, starvation, and response to pathogen’s infection. During the metamorphosis, autophagy is fiercely induced in the larval tissues, such as midgut, fat body, and salivary gland in insects (Tian et al., 2013; Romanelli et al., 2016; Nandy et al., 2018; Tettamanti and Casartelli, 2019). Inhibition of autophagy prevents the differentiation of silkworm stem cells and the formation of pupal epithelium during the larval-pupal metamorphosis in silkworm (Salasc et al., 2016). Moreover, appropriate enhancement of autophagy prolongs the life span of *D. melanogaster* (Yang et al., 2015; Su et al., 2019). The roles of autophagy in the replication of different microorganisms are usually pathogen-specific or host-specific. In *Drosophila*, bacteria *Listeria monocytogenes*, *Mycobacterium marinum*, *Salmonella enterica*, *Escherichia coli*, and *Wolbachia* all can be cleared by autophagy (Kuo et al., 2018). *Drosophila* PGRP-LE (peptidoglycan recognition protein LE) induces LC3/Atg8-targeting autophagy to eliminate the infection of *Listeria monocytogenes* by recognizing the bacterial peptidoglycan in hemocytes (Kaneko et al., 2006; Yano et al., 2008). Moreover, RNAi of *DmAtg5*, *DmAtg7* or *DmAtg12* leads to an increase of pathogen and a decrease in survival rate in *Drosophila* after *E. coli* infection, showing the positive role of autophagy in anti-bacteria (Ren et al., 2009). Notably, infection of vesicular stomatitis virus causes inhibition of Akt signaling, thereby activation of antiviral autophagy, to protect animals from viral lethality in *Drosophila* (Dong and Levine, 2013). Similarly, infection of Zika virus (ZIKV) induces the expression of *STING* (*Stimulator of interferon genes*), the component of NF-κB-dependent inflammatory signaling, to resist the replication of ZIKE by inducing autophagy in *Drosophila* neurons, while the precise mechanism of *STING* in inducing autophagy is not fully illustrated (Liu et al., 2018; Liu and Cherry, 2019). In contrast some viruses can escape from or utilize autophagy for viral proliferation. In *Aedes aegypti*, venom allergen-1 (AaVA-1), the female saliva-specific protein, intracellularly interacts with autophagy inhibitory protein LRPPRC (leucine-rich pentatricopeptide repeat-containing protein and the negative binder of Beclin-1), and thus upregulating autophagy to promote the transmission of mosquito-borne viruses, such as dengue fever and ZIKV in host (Sun et al., 2020). Of note, plant reovirus and rice gall dwarf virus induce the formation of autophagosomes for delivering virion, and thereby promotion of viral spread and transmission in *Recilia dorsali* (Chen et al., 2017). In *B. mori*, the infection of nucleopolyhedrovirus (BmNPV) induces autophagy to facilitate the viral replication mainly by upregulation of *BmAtg7* and *BmAtg9* (Wang et al., 2017).

Autophagy is simultaneously regulated at transcriptional and post-translational levels. TFEB (Transcription factor EB), FOXO (Forkhead box O), and primary-responsive transcription factors of 20-hydroxyecdysone (20E) signaling, as well as non-coding RNAs all affect the expression of *Atg* genes in insects (Demontis and Perrimon, 2010; Tian et al., 2013; Dai et al., 2020). In addition, the post-translational modifications including phosphorylation and acetylation of autophagy associated proteins mediate the occurrence. 20E and juvenile hormone (JH) synergistically regulate molting and metamorphosis of insects (Tian et al., 2013; Tettamanti and Casartelli, 2019). 20E induces while JH inhibits autophagy by antagonizing 20E signals (Liu et al., 2015; Tettamanti and Casartelli, 2019). Of note, the transcriptional and post-translational regulators of autophagy are usually linked, e.g., 20E signaling induces the expression of *Atg* genes and inhibits acetylation of Atg proteins simultaneously; moreover, 20E also interacts with nutrient signaling to initialize autophagosome formation (Tian et al., 2013; Wu et al., 2021a). Here, we summarize the most recent progresses of autophagy mainly in *D. melanogaster* and *B. mori* to provide a deep thinking for further autophagy-related studies in insects.

## TranscriptionAL and Post-transcriptional Regulation of Autophagy in Insect

### Nutrient Signaling, Insulin-Akt/PI3K-MTOR

The PI3K/Akt/MTOR signaling is critical for autophagy initiation by integrating signals from the extra- and intracellular environments (Wang et al., 2019). MTORC1 is well studied for inhibiting autophagy induction in insects (Tian et al., 2013; Liu et al., 2014; Dai et al., 2020). In *Drosophila*, MTORC1 affects RNA processing of *Atg* transcripts and thereby the protein levels, in addition to directly modulating phosphorylation status of DmAtg1/DmAtg13 protein complex (Tang et al., 2018). Under nutrient-rich conditions, MTORC1 decreases the protein levels of cyclin-dependent kinase 8 (CDK8) and darkener of apricot (DOA) kinase. When MTORC1 activity is inhibited by starvation, the restored CDK8 and DOA kinase lead to phosphorylation of CPSF6, the component of the cleavage and polyadenylation (CPA) complex of pre-RNAs, and thus nuclear translocation of the complex. Subsequently, the nuclear-localized CPA complex results in alternative polyadenylation of *DmAtg1* and *DmAtg8a*, and alternative splicing of *DmAtg1* transcript, as well as promotion of autophagy (Tang et al., 2018).

MTOR activity is affected by multiple factors in insects. Curcumin, a natural insecticide, reduces PI3K levels in a time-dependent manner and significantly compromises the protein levels of phosphorylated Akt and MTOR, which eventually leads to an increase in autophagy (Veeran et al., 2019). Glycogen synthase kinase 3 beta (GSK-3β) phosphorylates endonuclease G (ENDOG), a mitochondrial nuclease, to enhance its interaction with 14-3-3γ, thereby inhibiting MTOR signaling and promoting autophagy (Wang et al., 2021b). Downregulation of TGFB-INHB/activin-like protein daw activates TORC2 signaling to induce cardiac autophagy and reduces age-related heart dysfunction in *Drosophila* (Chang et al., 2020). Studies show that loss of ubiquitin causes endoplasmic reticulum stress, inhibition of MTORC1 activity, and promotion of autophagy, which consequently leads to neuronal death. Meanwhile, knockout of *Ubqn* (*ubiquilin*) reduces lysosomal acidification and autophagic flux, showing the requirement of ubiquitin in maintaining the proper levels of V0a/V100 subunit of Vacuolar H(+)-adenosine triphosphatases (V-ATPases; Şentürk et al., 2019). In general, as the center of nutrient signaling, MTOR cooperates with multiple signals to regulate the induction of autophagy.

### 20E Signaling in Regulating Autophagy

20E, the steroid hormone biosynthesized from food-derived cholesterol, predominantly orchestrates the physiological processes of molting, metamorphosis, and reproduction in association with nutrient and juvenile hormone signals in insects (Liu et al., 2015; Song and Zhou, 2020). During larval-pupal metamorphosis, 20E not only triggers the morphological changes of larvae to shed the old cuticle, but also induces the destruction of most larval tissues to recycle constituents for the formation of adult organs through activation of autophagy, apoptosis, and tissue dissociation (Xie et al., 2016; Jia et al., 2017). 20E transduces its signal by binding with the heterodimer receptor EcR-USP to form the ligand-receptor complex, 20E-EcR-USP, subsequently triggers the expression of the downstream transcription factors, such as *Br-C*, *E74*, *HR3*, and *βftz-F1*, which induce the cascades of *Atg* genes including *BmAtg1*, *BmAtg3*, *BmAtg4*, *BmAtg5*, *BmAtg6*, *BmAtg7*, *BmAtg8*, *BmAtg9*, *BmAtg12*, *BmAtg16*, and *BmAtg18*, and thereby autophagy (Tian et al., 2013). RNAi of *BmAtg1* blocks autophagy in the fat body during larval-pupal metamorphosis, when 20E titer is high in the hemolymph. Moreover, an *EcR* response element (EcRE) is identified in the promotor regions of *BmAtg1*, showing the direct transcriptional induction of *Atg* genes by 20E signaling (Tian et al., 2013). Notably, the zinc-finger transcription factor E75 and the helix-turn-helix (HTH) transcription factor E93 are both necessary for autophagy occurrence and larval-pupal metamorphosis in *B. mori* (Li et al., 2015, 2016a; Liu et al., 2015). In addition, E93 upregulates the expression of almost all *Atg* genes in both *Bombyx* and *Drosophila* to promote autophagy, while JH represses the transcription of *Bombyx BmE93*, suggesting a negative role of JH in autophagy induction through interaction with 20E signals (Liu et al., 2014, 2015). Of note, 20E signal also induces the transcription of *BmV-ATPase* from V0 and V1 subunits to acidify lysosomes and enhances autophagic flux in *B. mori* (Dai et al., 2020). Moreover, 20E can induce a starvation-like conditions and reduces the activity of Insulin-Akt/PI3K-MTORC1 pathway to initiate autophagy in insects (Tian et al., 2013; Liu et al., 2015).

**Figure 1:**
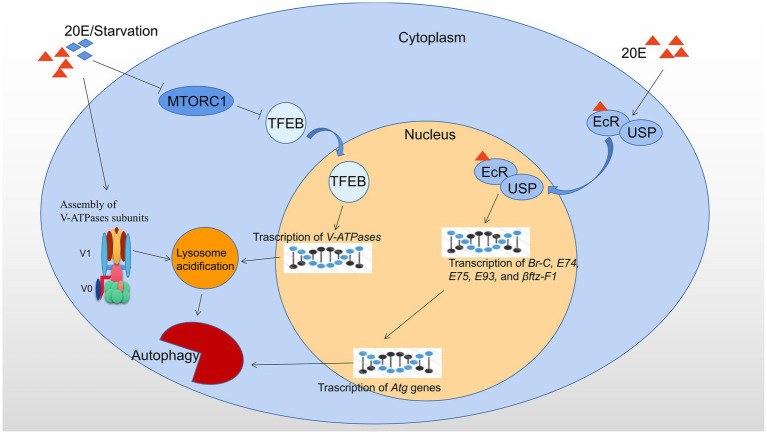
Transcriptional regulation of autophagy by 20E and starvation in *B. mori* and *D. melanogaster*. 20E and starvation both result in the cytoplasmic-nucleo translocation of TFEB by inhibiting MTORC1 activity and promote the assembly of V-ATPase subunits. Consequently, nuclear-localized TFEB promotes the transcription of *V-ATPases*, and thus upregulation of lysosomal acidification and autophagic flux. 20E forms a ligand-receptor complex with the heterodimer EcR/USP and then is transferred into the nucleus to initiate the expression of the downstream transcription factors, such as *Br-C, E74, E75, E93*, and *βftz-F1*, which subsequently leads to upregulation of *Atg* genes and autophagy. Red triangles indicate 20E; blue rhombuses indicate starvation.

The major regulators of autophagy at transcriptional level in *B. mori* and *D. melanogaster* are showed in Figure 1.

### Forkhead Box O, FOXO

Forkhead box O is an important transcription factor that plays a key role in the processes of cell metabolism, apoptosis, cell cycle, and stress response (Akasaki et al., 2014). FOXO1 and FOXO3a are reported to regulate the expression of *Atg* genes and activate autophagy in mouse (Li et al., 2016b). In *Drosophila*, FOXO and its targeting gene *4E-BP* upregulate autophagy to degrade damaged proteins, thereby delaying muscle functional decay and prolonging life span of *Drosophila* (Demontis and Perrimon, 2010).

In *Helicoverpa armigera*, insulin upregulates the expression of *phosphoinositide-dependent kinase-1* (*PDK1*) to promote phosphorylation of Akt. Subsequently, the phosphorylated Akt further phosphorylates FOXO and arrests FOXO in the cytoplasm, resulting in a high titer of 20E. In turn, the high 20E titer inhibits *PDK1* mRNA levels, and thus inhibition of Akt and FOXO phosphorylation, which leads to the nuclear localization of FOXO and thus induction of autophagy (Pan et al., 2018). Moreover, the production of 20E upregulates the expression of *PTEN* to remove Akt-mediated phosphorylation of FOXO, and leads to nuclear translocation of FOXO (Cai et al., 2016). In *B. mori*, 20E induces the transcription of *BmFOXO* and cytoplasmic-nucleo translocation of the protein (Hossain et al., 2013). Infection of BmNPV decreases the expression of *BmFOXO* in *B. mori*. Of note, RNAi of *BmFOXO* increases the viral replication, in comparison, overexpression of *BmFOXO* significantly increased the expression of autophagy-related genes *BmAtg6*, *BmAtg7*, and *BmAtg8*, and thereby inhibition of BmNPV duplication (Kang et al., 2021). In general, the nuclear localization and activity of FOXO are coordinately regulated by nutrient and 20E signaling in insects.

### Transcription Factor, TFEB

Transcription factor EB, a basic helix loop helix (b-HLH) leucine zipper transcription factors (MiT/TFE) from microphthalmia-associated family, is found to mediate autophagic flux and lysosome biogenesis, particularly in the expression of genes related to lysosomal acidification (Settembre et al., 2011; Song et al., 2013). In *Drosophila*, *MITF* is necessary for starvation-induced autophagy. MITF is transferred into the nucleus to induce the expression of genes encoding *V-ATPase*s, when MTORC1 activity is inhibited. Knockout of *MITF* causes lysosomal dysfunction and inhibits autolysosome maturation. In comparison, overexpression of *MITF* increases lysosomes, autophagosomes, and autolysosomes (Bouché et al., 2016). In *B. mori*, 20E and starvation induce lysosomal acidification by upregulating the transcription and assembly of V-ATPase subunits through activating TFEB by inhibiting MTOR activity (Dai et al., 2020).

Besides, some metabolites and molecular components are also involved in the regulation of TFEB activity. α-ketoglutarate (AKG), an important intermediate metabolite in the tricarboxylic acid (TCA) cycle, increases the expression of *TFEB* by activating AMPK and inhibiting MTOR signals, which subsequently leads to significant upregulation of *DmAtg1*, *DmAtg5*, *DmAtg8a*, and *DmAtg8b* in *Drosophila* (Su et al., 2019). Notably, the general control non-repressed protein 5 (GCN5) inhibits autophagy and lysosome biogenesis by catalyzing the acetylation of TFEB to abolish its DNA-binding ability in *Drosophila*. Interference of GCN5-mediated acetylation of TFEB increases autophagy and reduces aggregation of Tau protein, as well as its neurotoxicity in *Drosophila* (Wang et al., 2020b). In general, TFEB activity is modulated by several different signaling pathways and plays a critical role in autophagy.

### Other Transcription Factors

NF-κB factor Relish, a molecular component of immune deficiency (Imd) pathway, is essential for autophagy occurrence and tissue degradation triggered by steroid hormone 20E in the salivary glands after pupation in *Drosophila*. 20E induces the expression of *PGRP-LC* and its downstream gene *Relish*, which subsequently triggers the transcription of *DmAtg1* and activation of autophagy in *Drosophila* salivary glands (Nandy et al., 2018). Similarly, TmRelish mediates the upregulation of *TmAtg1* in response to the infection of *Listeria monocytogenes* in *Tenebrio molitor* (Keshavarz et al., 2020).

In *Drosophila*, nuclear factor (erythrocyte-derived 2)-related factor 2 (NRF2/ CncC), a member from the Cap'n' Collar (Cnc) transcription factor family, directly binds to the antioxidant response element in the promoter of *DmRef(2)P/p62*, a selective autophagy receptor, to induce its transcription upon oxidative stress, and thus playing a positive role in regulating autophagy, whereas CncC upregulates Atg8a protein level and autophagy independent of TFEB/MitF in *Drosophila* fat body and larval gut (Jain et al., 2015).

FOXA, the winged-helix transcription factors, plays key roles in organ-specific gene expression in the mammalian pancreas, liver, and dopaminergic neurons (Fox et al., 2013). *Fork head* (*Fkh*), the sole *Drosophila FOXA* family member, is proved to upregulate the expression of *Atg17*, the component of Atg1 kinase complex for autophagy initiation, in response to the decrease of insulin/insulin-like growth factor signaling (IIS) and significantly extends life span of *Drosophila*. Interestingly, overexpression of *Atg17* in neurons alone is sufficient to extend *Drosophila* life span, suggesting that autophagy is a beneficial effect downstream of neuronal *Fkh* to increase the health of life (Bolukbasi et al., 2021).

Of note, *Drosophila* transcription factor M1BP is functionally conserved with the homolog ZKSCAN3 of vertebrate (zinc-finger family DNA-binding protein) in inhibiting autophagy. RNAi of *M1BP* in *Drosophila* restores the transcription of *Atg* genes including *Atg1*, *Atg3*, *Atg7*, and *Atg8a*, which is inhibited by *ZKSCAN3* overexpression; in comparison, *M1BP* overexpression in HeLa cells prevents starvation-induced autophagy causing by nucleo-cytoplasmic translocation of ZKSCAN3, indicating the evolutionarily conserved repression of M1BP and ZKSCAN3 on autophagy from insects to mammals (Barthez et al., 2020).

### MicroRNA and lncRNA

MicroRNA (miRNA), a non-coding RNA composed of 19 to 25 nucleotides, inhibits the translation of target mRNA by pairing with the complementary sequence in the 3′UTR of target gene and also impairs the stability of mRNA in some cases (Bizuayehu and Babiak, 2014; Abo-Al-Ela and Burgos-Aceves, 2021). Several non-coding RNAs are reported to mediate autophagy occurrence in insects. In *Drosophila*, miR-14 is necessary for developmentally regulated process of tissue-specific autophagy in salivary gland but is dispensable for starvation-induced autophagy in fat body. miR-14 positively regulates autophagy by targeting the 3′UTR of *ip3k2*, a gene involved in inositol 1,4,5-triphosphate (IP3) signaling and calcium release from endoplasmic reticulum (ER). Particularly, miR-14 downregulates the translation of *ip3k2* and then increases the level of IP3. Subsequently, increased IP3 leads to the release of calcium and activates calmodulin, and thereby promotion of autophagy (Nelson et al., 2014). Screening through the miRNA library, miR-9 family is found to act as inhibitors of the phenotype induced by human *Tau* overexpression in *Drosophila*. Notably, miR-9a targeting gene *Drosophila CG11070* or its mammalian ortholog *UBE4B* (*ubiquitination factor E4B*) promotes autophagy-mediated Tau degradation in *Drosophila* in cooperation with STUB1 (STIP1 homology and U-Box containing protein 1), an E3/E4 ubiquitin ligase (Subramanian et al., 2021). The Hippo/Warts pathway functions as the key tumor suppressor by limiting organ growth. In response to insulin signaling, Warts inactivates Yorkie to inhibit the expression of downstream effector miRNA *bantam* and positively regulates the production of ecdysone in prothoracic gland (PG) of *Drosophila* by induction of autophagy. In addition, PG-specific overexpression of miRNA *bantam* activates mTOR and reduces the protein level of EcR, leading to suppression of autophagy and decrease of ecdysone production by limiting the availability of the precursor cholesterol (Texada et al., 2019).

Long non-coding RNA (lncRNA) is a series of RNA with a length of more than 200 nucleotides without the ability to encode proteins (Li et al., 2021). The mechanism of lncRNA in regulating autophagy has been wildly studied in mammals while less in insects. A recent study shows that 20E significantly increases the expression of lncRNA *LNC_000560* in *B. mori* larvae. Moreover, the expression profile of *LNC_000560* is highly consistent with that of *BmAtg4b*, and RNAi of *LNC_000560* significantly reduces the expression of *BmAtg4b*. These results indicate that *LNC_000560* is associated with 20E-induced autophagy in the silkworm fat body through positive regulation of *BmAtg4b* (Qiao et al., 2021).

## Post-translational Regulation of Autophagy in Insects

### Phosphorylation

Autophagic function of ULK1/Atg1-ATG13/Atg13 protein complex is regulated by their phosphorylation status (Klionsky et al., 2021). In *Drosophila* and mammals, ULK1/Atg1 and Atg13/ATG13 form a stable complex regardless to the status of autophagy occurrence; whereas in yeast, Atg1 binds to Atg13 only under the induction of autophagy (Chang and Neufeld, 2010; Li et al., 2020). Under nutrient-rich conditions, MTOR inhibits the formation of Atg1 complex by hyperphosphorylation of Atg13, and thereby blockage of its interaction with Atg1 and autophagy in yeast (Puente et al., 2016). In mammals, starvation reduces phosphorylation level of ATG13 to upregulate autophagy (Zachari and Ganley, 2017). Whereas in *Drosophila*, DmAtg13 is highly phosphorylated, while DmAtg1 is partly dephosphorylated during autophagy induction (Kim et al., 2013; Nagy et al., 2014). Of note, two bands of BmAtg13 appear in the western blot detection, and the upper band is impaired after 20E treatment. It suggests that BmAtg13 may be phosphorylated under nutrient-rich conditions while dephosphorylated during 20E- or starvation-induced autophagy in *B. mori* (Li et al., 2020).

In addition to Atg proteins, phosphorylation also influences the autophagic functions of other protein in insects. BmRpd3/BmHDAC1, the homolog of human histone deacetylase HDAC1 in *B. mori*, localizes in the nucleus under nutrient-rich conditions but is transferred to the cytoplasm to promote autophagy in response to 20E signaling. Overexpression of *BmHDAC1* promotes the formation of BmAtg8–PE, degradation of BmSqstm1, and lysosomal acidification. Notably, cholesterol and its derivatives, such as 20E and 27-hydroxycholesterol, all induce dephosphorylation of BmHDAC1 by inhibiting the activity of MTORC1, leading to nucleo-cytoplasmic translocation of BmHDAC1 and promotion of autophagy. The regulatory mechanism of BmHDAC1 and its human homolog HsHDAC1 by cholesterol/20E signaling is highly conserved, and potentially, from insects to mammals (Wu et al., 2021b). In *Drosophila*, Cdk5, a cyclin-dependent kinase, phosphorylates Acn/Acinus (a multifunctional nuclear protein with proposed roles in apoptosis) at serine 437 to regulate its stability. Replacements of Acn protein by the expression of nonphosphorylatable Acn^S437A^ or phosphomimetic *Acn^S437D^* under the control of endogenous promoter of *Acn* significantly decrease or increase the protein level. In addition, *Acn^S437D^* overexpression increases the basal and starvation-independent autophagy (Nandi and Kramer, 2018). Infection of rice black-streaked dwarf virus (RBDSV) or overexpression of *RBSDV P10* (the main capsid protein of RBSDV) promotes phosphorylation of AMPK in *Laodelphax striatellus* or in *Spodoptera frugiperda* Sf9 cells, which consequently leads to further phosphorylation and cytoplasmic-nucleo translocation of GAPDH, accompanied with activation of autophagy and inhibition of viral replication (Wang et al., 2021c).

### Acetylation

In *D. melanogaster*, brain-specific knockdown of AcCoAS/acetyl-coenzyme A synthetase enhances autophagy and prolongs life span, indicating that protein acetylation is involved in the regulation of autophagy (Eisenberg et al., 2014; Wani et al., 2015). *Drosophila* YL-1 and sirt2 oppositely catalyze the deacetylation and acetylation of DmAtg8a under starvation and nutrient-rich conditions. After deacetylation, DmAtg8a binds to DmSequoia, a negative transcriptional regulator of autophagy, in a LC3-interacting region motif-dependent manner, and consequently compromises the inhibition of autophagy by DmSequoia (Jacomin et al., 2020). Up to now, the regulatory mechanism of autophagy by acetylation is not fully elucidated in *Drosophila*.

In *B. mori*, 20E and its precursor cholesterol both induce dephosphorylation of BmHDAC1 and its nucleo-cytoplasmic translocation to promote autophagy, while the functional mechanism of BmHDAC1 in regulating autophagy is lack of investigation (Wu et al., 2021b). It is reported that the lysine site K13 of BmAtg8 protein is acetylated after BmNPV infection, and acetylation-mimic mutation of K13 in BmAtg8 significantly reduces autophagy (Xue et al., 2019). In order to fully understand the regulation of autophagy by acetylation in silkworm, an in-depth study of the molecular components BmAtg3, BmAtg4, BmAtg7, and BmAtg8 from BmAtg8–PE ubiquitin-like protein system has been performed. Results show that all the four aforementioned Atg proteins localize in the nucleus under nutrient-rich conditions, while they are exported from the nucleus to the cytoplasm during autophagy induction. In addition, histone acetyltransferase BmP300 and deacetylase BmHDAC1 oppositely regulate the nuclear and subcellular localization of BmAtg3 and BmAtg8. BmHDAC1 can deacetylate BmAtg3, BmAtg4, BmAtg7, and BmAtg8. Loss-of-function mutations of the acetylation sites lead to deacetylation and nucleo-cytoplasmic translocation of the four Atg proteins, and thereby promotion of autophagy. Collectively, acetylation/deacetylation of the molecular components from BmAtg8–PE ubiquitin-like protein system catalyzed by P300/HDAC1 mediates the occurrence of autophagy and is highly conserved from insects to mammals (Wu et al., 2021a).

**Figure 2:**
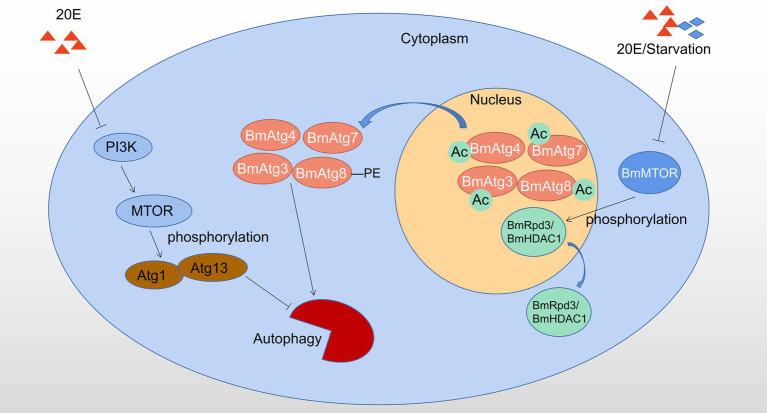
Autophagy regulated by phosphorylation and acetylation in *Bombyx mori* and *Drosophila melanogaster*. 20E affects the phosphorylation status of Atg1-Atg13 complex by inhibiting PI3K-MTOR activity to induce autophagy; 20E or starvation signal causes dephosphorylation of BmRpd3/ BmHDAC1, which subsequently leads to deacetylation and nucleo-cytoplasmic translocation of the molecular components from BmAtg8 -PE ubiquitin-like protein system, thereby promotion of autophagy, Ac: acetylation. Red triangles indicate 20E; blue rhombuses indicate starvation.

Autophagy regulated by post-translational modifications in *B. mori* and *D. melanogaster* is showed in Figure 2.

## Conclusion

Transcription factors, such as FOXO, TFEB, and Relish, as well as those involved in 20E signal transduction, are reported to upregulate autophagy in insects. Some of them are functionally conserved to the mammalian homologs, while some of them act in insects specifically. 20E, the insect-specific regulator, predominantly upregulates autophagy by inducing the expression of almost all *Atg* genes directly; in addition, 20E signaling also interacts with other transcription factors, such as FOXO and Relish, to regulate autophagy at transcriptional level indirectly. 20E promotes the transcription of *FOXO* by binding to the promotor region and simultaneously induces the dephosphorylation of FOXO, which consequently leads to cytoplasmic-nucleo translocation and upregulation of autophagy (Hossain et al., 2013; Cai et al., 2016). As mentioned above, 20E activates the transcription and nuclear translocation of BmTFEB, which consequently triggers the cascades of *BmV-ATPase* expression as well as assembly of the subunits, and thus promotion of lysosomal acidification and autophagic flux (Dai et al., 2020). In general, 20E affects the expression and PTM (post-translational modification) status of the transcription factors to promote their positive functions in autophagy occurrence, accompanying with its direct induction of *Atg* and *V-ATPases* gene expression in insects. To our knowledge, there are more transcription factors involved in regulating autophagy in plants, yeast, or mammals, whereas they have not been illustrated in insects and wait for further investigation.

Phosphorylation and acetylation are well-studied PTMs in regulating autophagy in insects. Mammalian HDAC1 is previously reported to induce *Atg* gene expression and autophagosome formation, thereby promoting autophagy (Moresi et al., 2012). Cholesterol and its derivatives, such as 20E, induce dephosphorylation of histone deacetylase *Bombyx* BmHDAC1 and the human homolog HsHDAC1 to facilitate autophagy by inhibition of MTOR activity (Wu et al., 2021b). In addition, the acetylation levels of Atg proteins from LC3/BmAtg8–PE ubiquitin-like protein system are increased by histone acetyltransferase P300 while decreased by deacetylase HDAC1 in *B. mori* and human. Subsequently, deacetylation of the Atg proteins mediated by HDAC1 leads to their nucleo-cytoplasmic translocation and autophagy occurrence (Popelka and Klionsky, 2015; Wu et al., 2021a). These results show that the regulatory mechanism of acetylation in regulating autophagy is highly conserved in *B. mori* and mammals (Wu et al., 2021b). Whereas knockout of yeast deacetylase *RPD3*, the homolog of mammalian and *Bombyx* HDAC1, leads to premature autophagy, in comparison, overexpression of yeast acetyltransferase *Esa1* promotes autophagy by catalyzing the hyperacetylation of Atg3 (Yi et al., 2012). It is likely that the function of HDAC1 homolog in regulating autophagy is conserved from insects to mammals, but not in yeast. Of note, in addition to phosphorylation and acetylation, several other PTMs, such as ubiquitination, SUMOylation, glycosylation, and lipidation (PEylation of LC3/Atg8), are revealed to regulate autophagy in yeast and mammals, whereas, these PTMs have not been studied for their autophagic functions in insects and are worthy of further investigation (Popelka and Klionsky, 2015; Wani et al., 2015).

## Author Contributions

LT and KL conceived, revised, and edited the manuscript. RL, YX, and LT collected the studies and drafted the manuscript. All authors contributed to the article and approved the submitted version.

## Funding

This work is supported by the Natural Science Foundation of China (NSFC, 31970463 for LT, 32070491 for KL, and 31802135 for YX), Laboratory of Lingnan Modern Agriculture Project (NZ2021021 for LT), and Natural Science Foundation of Guangdong Province for LT (2017A030311024).

## Conflict of Interest

The authors declare that the research was conducted in the absence of any commercial or financial relationships that could be construed as a potential conflict of interest.

## Publisher’s Note

All claims expressed in this article are solely those of the authors and do not necessarily represent those of their affiliated organizations, or those of the publisher, the editors and the reviewers. Any product that may be evaluated in this article, or claim that may be made by its manufacturer, is not guaranteed or endorsed by the publisher.
